# Functional Limitations, Medication Support, and Responses to Drug Costs among Medicare Beneficiaries

**DOI:** 10.1371/journal.pone.0144236

**Published:** 2015-12-07

**Authors:** Christopher Whaley, Mary Reed, John Hsu, Vicki Fung

**Affiliations:** 1 School of Public Health, U.C. Berkeley, University of California, Berkeley, CA, United States of America; 2 Division of Research, Kaiser Permanente Northern California, Oakland, CA, United States of America; 3 Mongan Institute for Health Policy, Massachusetts General Hospital, Boston, MA, United States of America; 4 Department of Medicine Harvard Medical School, Boston, MA, United States of America; 5 Department of Health Care Policy, Department of Health Care Policy, Boston, MA, United States of America; University of Miami School of Medicine, UNITED STATES

## Abstract

**Objective:**

Standard Medicare Part D prescription drug benefits include substantial and complex cost-sharing. Many beneficiaries also have functional limitations that could affect self-care capabilities, including managing medications, but also have varying levels of social support to help with these activities. We examined the associations between drug cost responses, functional limitations, and social support.

**Data Sources and Study Setting:**

We conducted telephone interviews in a stratified random sample of community-dwelling Medicare Advantage beneficiaries (N = 1,201, response rate = 70.0%). Participants reported their functional status (i.e., difficulty with activities of daily living) and social support (i.e., receiving help with medications). Drug cost responses included cost-reducing behaviors, cost-related non-adherence, and financial stress.

**Study Design:**

We used multivariate logistic regression to assess associations among functional status, help with medications, and drug cost responses, adjusting for patient characteristics.

**Principal Findings:**

Respondents with multiple limitations who did not receive help with their medications were more likely to report cost-related non-adherence (OR = 3.2, 95% CI: 1.2–8.5) and financial stress (OR = 2.4, 95% CI: 1.3–4.5) compared to subjects with fewer limitations and no help; however, those with multiple limitations and with medication help had similar odds of unfavorable cost responses as those with fewer limitations.

**Conclusion:**

The majority of beneficiaries with functional limitations did not receive help with medications. Support with medication management for beneficiaries who have functional limitations could improve adherence and outcomes.

## Introduction

As the U.S. population ages, the number of Americans living with disability and functional limitations is expected to increase [1]. The demographic shifts are occurring within the context of substantial medical spending constraints in which the Medicare program is unable to sustain current rates of spending growth and will exhaust its trust fund by 2026 [2]. Beneficiaries with functional limitations often have greater clinical needs and higher medical spending. They may also face a greater burden from cost-sharing, which is a commonly used approach by payers, including Medicare, to control spending. Moreover, there is wide variability in the level of social support and assistance many older adults receive with personal and home activities, but little is known about the associations between functional limitations, medication assistance, and beneficiaries’ responses to drugs costs.

Patient cost-sharing is associated with reductions in both necessary and unnecessary medical care in the general population [3–7] and has been associated with reductions in chronic drug use and worse clinical outcomes in the Medicare Part D prescription drug program [8–11]. However, little evidence exists about the effects of Part D cost-sharing on medication management activities for beneficiaries with limited functional status. Beneficiaries with functional limitations or disability could face greater challenges navigating responses to out-of-pocket drug costs compared with those without limitations due to cognitive or physical impairments, such as due to challenges associated with understanding benefits or accessing or communicating with providers [12].

Most caregiving comes from unpaid, informal sources, such as family members or friends [13], and levels of unmet need for assistance with daily living among the elderly are still substantial, especially for those with multiple functional limitations [14]. Nevertheless, caregiving and social support have been linked to improved medication adherence and outcomes among individuals with chronic conditions [15–20]. Few studies, however, have assessed the specific types of assistance that beneficiaries with functional limitations receive with medications and who provides this assistance. In addition, while several studies examine the role of cost-sharing and functional limitations on medication behavior independently, no studies examine whether the availability of medication assistance is associated with the relationship between functional limitations and responses to drug costs.

This study investigates these questions in a sample of Medicare Advantage beneficiaries in a large integrated delivery system using data from telephone interviews. We hypothesized that beneficiaries with multiple functional limitations would be more vulnerable to potentially adverse responses to cost-sharing, including cost-related non-adherence and financial stress. We also hypothesized that the availability of medication assistance could mitigate these adverse effects. We focused explicitly on well-defined functional limitations and assistance with medications, while recognizing that there are many other potential factors that could increase individuals’ vulnerability to adverse health events, and that these traits often occur together.

## Methods

### Study Design and Population

All participants were continuously enrolled in the Kaiser Permanente Northern California (KPNC) Medicare Advantage (MA) plan throughout 2008 and were age 65 years or older as of January 1, 2008. KPNC is an integrated delivery system providing comprehensive care to over three million members, including approximately 300,000 Medicare Advantage beneficiaries. The study included a stratified random sample of beneficiaries who were individual subscribers to the Medicare Advantage Part D plan, beneficiaries receiving Part D low income subsidies (LIS), and beneficiaries with employer-supplemented insurance (ESI) benefits. LIS and ESI beneficiaries have lower cost sharing for prescription drugs than the individual subscribers and do not have a Part D coverage gap. We also oversampled beneficiaries with high levels of drug spending in 2008; the details of the survey sample and protocol have been described elsewhere [7]. Because the focus on the study was on community-dwelling beneficiaries, we excluded dual-eligible beneficiaries eligible for both Medicare and Medicaid with evidence of institutionalization based on Medicare Monthly Membership Reports; reliable indicators of institutionalization were not available in the datasets for non-dual-eligibles, thus these individuals were screened at the time of the interview (see below). All analyses are weighted for sampling proportions to represent the target population of KPNC community-dwelling Medicare Advantage beneficiaries age 65 or older.

### Interview Protocol and Eligibility

We contacted a total of 2,650 potential respondents starting in January 2009. Respondents could decline participation via postcard or by phone, or complete the questionnaire and return it by mail. Trained interviewers contacted the remaining respondents by telephone, obtained informed verbal consent and administered the interview. Participants who were institutionalized, primarily those in nursing homes or assisted living facilities, or who were unable to complete the interview, e.g., due to language barrier, cognitive impairment, severe illness, or death were excluded. Those who left KPNC or for whom we did not have correct contact information were also excluded. Of the remaining 1,714 eligible participants, 1,201 completed the survey (response rate = 70.0%) [7]. The Kaiser Permanente Northern California Institutional Review Board approved the study.

### Survey Measures: Functional Limitations, Help with Medications, and Cost Responses

We assessed participants’ functional status by asking if, because of a health or physical problem, they had difficulty with any of six activities of daily living (ADLs)—bathing or showering, dressing, eating, getting in or out of bed or chairs, walking, and using the toilet—and six instrumental activities of daily living (IADLs)—using the telephone, doing light housework, doing heavy housework, preparing meals, shopping for personal items, and managing money. Because interviews were conducted over the telephone, this study likely underrepresents potential participants who report difficulty using the telephone.

We also asked participants if, “[d]uring 2008, did anyone, such as a friend or family member, help you with your prescription medications (such as filling prescriptions, paying for them, or deciding how to take them), or did you generally do these things on your own?” If participants reported receiving help, open-ended responses were used to assess who provided help and how help was provided. Among 183 participants reporting help with medications, 177 described who helped them and 149 described the kind of help they received. The open-ended responses from this question were independently coded into common types and sources of medication help by two researchers; a third researcher resolved disagreements. Many participants reported multiple sources and forms of medication help and so one respondent’s response could be coded into multiple themes.

Lastly, participants reported whether, because of the amount they had to pay for medications in the previous year, they engaged in cost-reducing behaviors (switching to generics and splitting or skipping pills according to doctor’s advice), cost-related non-adherence (skipping doses, not refilling prescriptions, or stopping the medication altogether due to cost and without a doctor’s advice), or experienced financial stress (borrowing money or cutting back on necessities to help pay for medications).

### Data Analysis

Multivariate logistic regression was used to assess associations between functional limitations, help with medications, and drug cost responses. To examine functional limitations, we used a binary definition for having multiple limitations: difficulty with 0–1 vs. 2+ ADLs or IADLs. In sensitivity analyses, we examined different categorizations (e.g., 0, 1–2, 3+ functional limitations) and the findings were similar. We stratified these two groups based on whether participants reported receiving help with medications, resulting in four total groups: 0–1 functional limitations without help with medications (reference group); 0–1 limitations with help; 2+ limitations without help; and 2+ limitations with help. The multivariate analyses also adjust for each subject’s coverage type (i.e., LIS, ESI or individual subscriber), 2008 drug spending, and gender and age, which were obtained from automated health plan databases. We also adjusted for characteristics obtained from the survey, including race, income, education, and number of medications prescribed in 2008 (5+ vs. < = 5). All analyses were weighted for sampling proportions (svy: logistic in Stata 10.1). We calculated the adjusted percentage of participants reporting each cost response in each of the functional status-help groups based on the model results. The standard population used in the direct adjustment procedure was the mix of covariable values in the overall target population of MA beneficiaries.

## Results

### Population Characteristics

Table 1 shows population characteristics stratified by the number of functional limitations. Those with multiple (2+) functional limitations tended to be older, had lower incomes and educational attainment, and were less likely to be living with a spouse or partner. They also were more likely to use five or more different medications and had higher annual drug spending in 2008. Finally, those with 2+ functional limitations were more likely to receive Medicare Part D low-income subsidies (LIS). The LIS population included dual-eligible beneficiaries with Medicaid, which substantially reduces cost-sharing requirements compared to the non-Medicaid eligible population.

**Table 1:** Population Characteristics.

# Total functional limitations (ADLs and IADLs)				
	0–1	2+	Total	*p-value*
*N*	*772*	*429*	*1*,*201*	
**Age**	%	%	%	
65–74	55.6	32.6	49.8	<0.001
75–84	39	48.7	41.4	0.015
85+	5.4	18.7	8.8	<0.001
**Sex**				
Female	58.0	68.0	60.5	0.009
**Income**				
Below $40k	45.1	66.9	50.7	<0.001
**Race**				
White	79.2	75.0	78.1	0.197
**Education**				
High school diploma or higher	69.1	59.9	66.8	0.017
**Living with spouse or partner**				
Yes	66.8	45.0	61.3	<0.001
**Self-reported health**				
Excellent or very good	50.9	20.0	43.2	<0.001
**Drug spending**				
$0-$1,000	69.6	44.2	63.3	<0.001
$1,001-$2,510	21.5	32.8	24.4	<0.001
$2,510+	8.8	23	12.4	<0.001
**Used 5+ medications in last 12 months**				
Yes	35.1	62.4	41.9	<0.001
**Part D Coverage**				
Individual MA subscriber (coverage gap)	51	44.2	49.3	0.093
Employer-sponsored MA coverage (no gap)	45.3	46.1	45.5	0.837
Part D low-income subsidy (no gap)	3.6	9.7	5.1	<0.001

Note: All percentages weighted for sampling proportions. MA = Medicare Advantage. There were 1,201 total respondents.

### Functional Limitations

**Fig 1:**
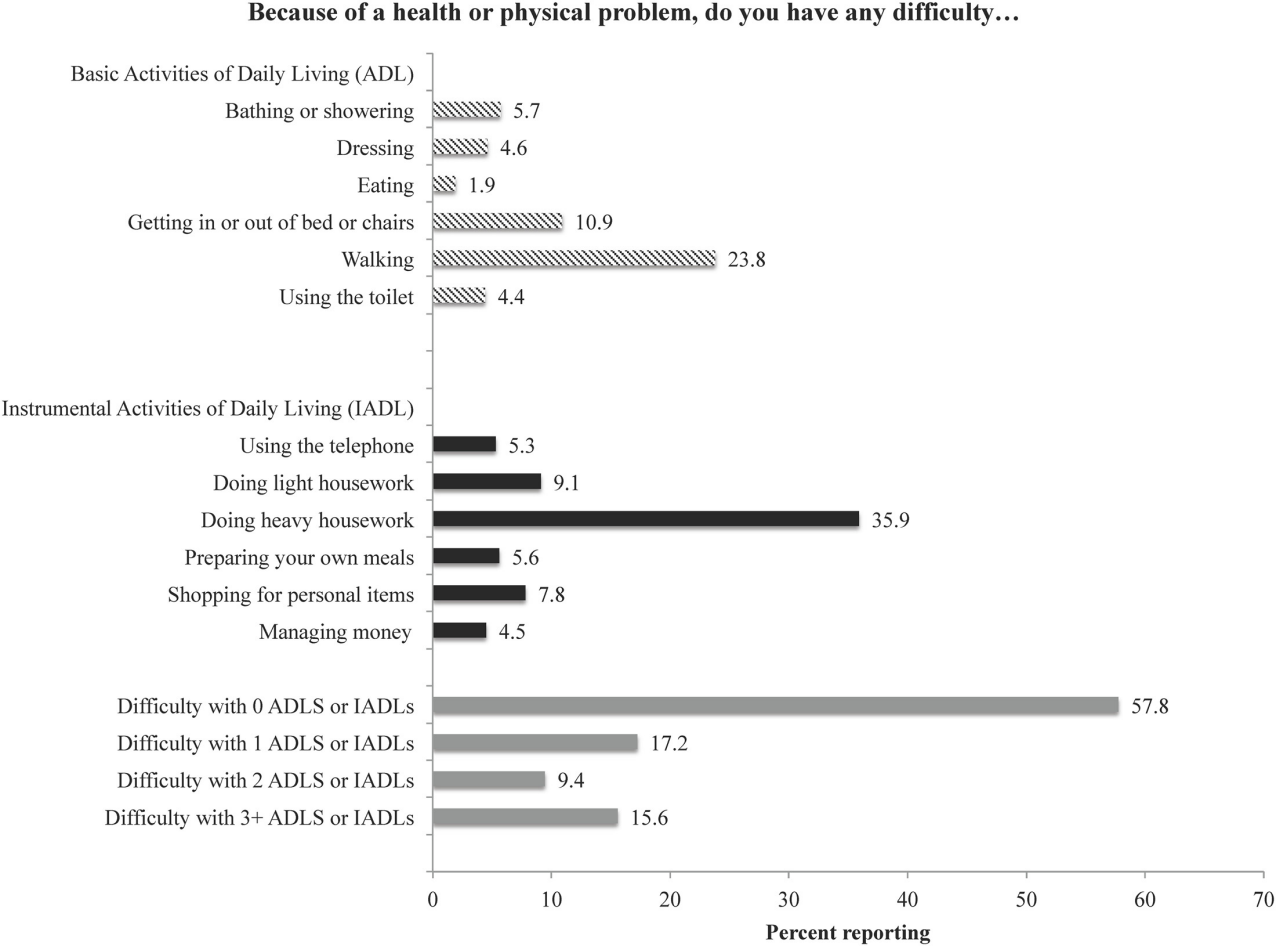
Percent of participants reporting difficulties with basic and instrumental ADLs. Note: Percentages weighted for sampling proportions; ADLs = Activities of Daily Living; IADLs = Instrumental Activities of Daily Living.

Among all participants, 57.8% of participants reported no functional limitations, 17.2% reported difficulty with 1 ADL or IADL and 25.0% reported difficulty with 2+ combined ADLs and IADLs (Fig 1). Additionally, 25.9% reported difficulty with one or more ADLs and 38.1% reported difficulty with one or more IADLs. The most common ADL limitations include walking (23.8%), and getting in or out of bed and/or chairs (10.9%). The most common IADL limitations include doing heavy housework (35.9%) and doing light housework (9.1%).

### Help with Medications

Overall, 9.5% of participants reported receiving help with their medications; 5.7% of those with 0–1 functional limitations received help compared with 20.6% of those with 2+ limitations (p<0.01, Table 2). Among those who received any help with their medications, the most common providers of medication assistance were from informal sources: spouses/partners (55.4%), children (33.7%), other family members (9.3%), or friends (4.2%). In contrast, 2.5% received medication help from caregivers in addition to family members, and 2.0% received help from caregivers only. Overall, 12.6% of those receiving help reported that they received help from multiple sources.

**Table 2:** Frequency and Types of Help with Medications.

**No. of functional limitations**	**% w/ Rx help** *	**p-value**
0–1	5.7%	<0.001
2+	20.9%	
**Who helps?**	**% Among those reporting help** *	**Key Example**
Spouse or partner	55.4%	Husband helps me with everything related to prescriptions, so I have no clue as to how much things cost. We never worried about it too much.
Child	33.7%	My son because he knows how to use the internet or phone call [order] and I don't know how at all.
Other family member	9.3%	Daughter-in-law calls them in and does all the stuff that needs to be done.
Caregiver	4.5%	My caregiver has occasionally picked prescriptions up for me at the pharmacy.
Friends	4.2%	A friend fills prescriptions, picks them up
**How do they help?**		
Fill or order prescriptions	38.6%	My husband does the re-orders and pick-ups on my Rx, and he does this all the time.
Pickup prescriptions	28.4%	My spouse, sometimes my kids and grandkids pick them up for me too. I don't walk so well.
Advise on how to take medications	21.3%	My daughter picks them up and explains to me how to take my medications.
Pay for medications	9.7%	My son helped me by paying for my medication when I could not afford it
Drive to pharmacy	2.5%	Daughter drives me and we both go in to the pharmacy to get the drugs.

*Weighted percent among those who report receiving help with medications (N = 183) who describe who provides help (N = 177) and what type of help is provided (N = 149). Many respondents listed multiple types of help so sums exceed 1.

Among those receiving help with their medications, the most common forms of help included filling or ordering (38.6%) and picking up (28.4%) prescriptions; 21.3% received advice on how to take medications, including receiving help administering medications, and 9.7% received help paying for medications. Among those receiving help, 47.4% reported that their caregivers provided multiple types of help: 43.9% and 51.1% (p = 0.478) among those with 0–1 and 2+ limitations, respectively.

### Responses to Drug Costs, Functional Status, and Help with Medications

In unadjusted analyses, 18.6% of participants engaged in a cost-reducing behavior, such as switching to a lower cost-drug (15.9% vs. 26.2% for those with 0–1 vs. 2+ functional limitations, p = 0.001); 3.1% experienced cost-related non-adherence, such as not refilling a drug due to costs (1.9% vs. 6.4% for those with 0–1 vs. 2+ functional limitations, p< 0.001). In addition, 8.6% reported financial stress, such as cutting back on a necessity to pay for medications (5.8% vs. 16.4% for those with 0–1 vs. 2+ functional limitations, p< 0.0001).

In multivariate analyses (Table 3), compared to those with 0–1 limitations without help with medications, those with 2+ limitations with no medication help were significantly more likely to experience cost-related non-adherence (OR = 3.22, 95% CI: 1.22–8.53) and financial stress (OR = 2.42, 95% CI: 1.29–4.54). In contrast, there were no significant differences between those with 2+ functional limitations who received help with their medications and those with 0–1 limitations in their odds of reporting any of the cost behaviors.

**Table 3:** Associations between drug cost-responses, functional limitations, and having help with medications.

	*adjusted percent*	*odds-ratio*	*95% CI*
**Cost-reducing behavior**			
0–1 functional limitations, **no** help with medications	16.9%	Ref	—
0–1 functional limitations, help with medications	16.9%	1.00	(0.38–2.64)
2+ functional limitations, **no** help with medications	22.9%	1.47	(0.89–2.42)
2+ functional limitations, help with medications	28.5%	2.00	(0.95–4.19)
**Cost-related non-adherence**			
0–1 functional limitations, **no** help with medications	1.9%	Ref	—
0–1 functional limitations, help with medications	5.1%	2.96	(0.76–11.58)
**2+ functional limitations, no help with medications**	**5.5%**	**3.22** *	**(1.22–8.53)**
2+ functional limitations, help with medications	1.1%	0.57	(0.15–2.15)
**Financial stress**			
0–1 functional limitations, **no** help with medications	6.5%	Ref	—
0–1 functional limitations, help with medications	9.9%	1.63	(0.60–4.41)
**2+ functional limitations, no help with medications**	**13.6%**	**2.42** **	**(1.29–4.54)**
2+ functional limitations, help with medications	11.0%	1.85	(0.83–4.11)

** p<0.01,

* p<0.05

95% CI in parentheses; Logistic regression weighted for sampling proportions and adjusted for age, sex, race, income, education, self-reported health, drug spending, number of medications, and Part D insurance coverage type.

## Discussion

This study investigated the prevalence of functional limitations, the availability of help with medications, and responses to drug costs in a population of community-dwelling Medicare Advantage beneficiaries. We found that one quarter of MA beneficiaries reported multiple functional limitations, defined as difficulty with two or more ADLs or IADLs. Among these beneficiaries, the majority did not have help with medications from family members, friends or caregivers. In multivariate analyses we found that potentially adverse responses to costs, including cost-related non-adherence and financial stress were most common among those with multiple functional limitations without help with medications. In other words, the lack of informal support was strongly associated with behaviors that are linked to adverse medical outcomes among a vulnerable group of elderly beneficiaries.

We found that fewer than one-in-ten beneficiaries reported receiving help with their medications in general and approximately one-in-five beneficiaries with multiple functional limitations received help. Family members, particularly spouses and children, provided the majority of help with medications, and most participants cited only a single source of assistance with medications. The most common types of assistance reported included basic tasks such as filling/ordering or picking up prescriptions. These types of assistance could be especially helpful for beneficiaries with mobility limitations, which were the most commonly reported limitations in this study. In addition, nearly half received help with multiple aspects of medication management and over 20% received help or advice in taking their medications.

Importantly, we found that potentially adverse effects of cost-sharing, including cost-related medication non-adherence and financial stress, were more common among beneficiaries with multiple functional limitations. However, for those who received help with medications, this association was weaker, suggesting that social support is potentially protective against some of the more unfavorable behaviors in response to drug costs. Improving medication adherence, especially for patients with multiple functional limitations, could improve health outcomes and reduce use of high-cost services due to unmet healthcare needs [21,22]. These findings underscore the importance of informing family members and including them in discussions about medication regimens, especially for elderly patients with functional limitations. A prior study found only 20% of informal caregivers received training or instructions for administering multiple medications and 12% reported making mistakes when administrating medications [23]. Greater involvement of caregivers in medication discussions could improve the quality of medication management and outcomes.

Of concern, the availability of informal caregiving is projected to decrease as the population ages, particularly to the extent that existing social trends persist with respect to household structure and geographic movement, which could result in an increasing number of beneficiaries with multiple functional limitations lacking needed assistance with medication management and other self-care activities [24]. As this trend continues, Medicare policies that promote and fund assistance with paying for or obtaining caregiving services are likely to grow in importance. Currently, Medicare provides home health care benefits to approximately 3.4 million beneficiaries; overall, Medicare spending on long-term care and home health services has increased substantially over recent years, from $29 billion in 2001 to $60 billion in 2012 [25]. To qualify for home health coverage, Medicare beneficiaries must be homebound. Until recently, Medicare required that a patient’s condition must be expected to improve and did not allow for continuous care [26]. A recent ruling in early 2013 could ease this restriction, making it easier for beneficiaries with permanent disabilities to obtain home health services; the impact of this change could manifest over years as information about the rule change has been slow to disseminate [27,28].

Nonetheless, home health benefits may not be a good substitute for informal sources of medication support that often includes assistance with basic tasks, such as filling or ordering medications. For example, patients could have differing levels of trust or confidence in paid home care staff than they do with family caregivers. Moreover, part-time or intermittent non-skilled home care or care for cognitively intensive activities including making financial decisions generally are not covered. Alternatively, some recent demonstration projects that provide high-risk and high-cost individuals with in-person assistance with self-care activities, including adherence to treatment recommendations, could be promising models for providing assistance with basic medication management tasks that contribute to medication adherence [29,30]. Our findings highlight the potential benefits of identifying effective and efficient models for providing these types of assistance.

## Limitations

This study has limitations to note. First, we focused on community-dwelling elderly Medicare Advantage beneficiaries in a large integrated delivery system who were able to complete the telephone survey, which could limit generalizability. Institutionalized beneficiaries, those who qualify due to disability (<age 65), and those with cognitive impairment or severe illness who could not complete the survey are likely to have worse functional status and higher drug costs, and potentially greater difficulty managing medications and responding to drug costs. However, we found similar levels of functional limitations (e.g., at least one ADL or one IADL limitation) in our study population compared with the existing literature. We also only measured difficulties with 12 common activities of daily living as a general proxy for functional, cognitive, and emotional limitations that could affect an individual beneficiary’s ability for self-care.

Similarly, we only collected information on limited types of support that beneficiaries might receive, i.e., the survey asked specifically about help with medications but not other types of social support or availability of caregiving. Thus, this study likely provides conservative estimates of the prevalence of clinically meaningful limitations, social support, and the potential interaction between the two on medication management compared with the general Medicare population [13]. In addition, this was a cross-sectional survey and we are thus unable to establish a causal relationship between medication assistance and behaviors. Although we were able to control for a large number of potential confounders available in health plan databases and the survey, including drug use and spending, drug coverage levels, health status/comorbidity levels, and demographic characteristics, such as household income and marital status, there could be remaining unobserved confounders.

Lastly, this survey was not designed to assess the clinical effects or clinical appropriateness of the drug cost responses; for example, beneficiaries could reduce adherence to less clinically necessary medications. These measures of cost-reducing behaviors, cost-related non-adherence and financial stress have been widely used in other studies, including national surveys [7,31–33].

## Conclusion

In a sample of elderly Medicare Advantage beneficiaries, we found that receipt of help with medications was limited, even among those with multiple functional limitations. The frequency of drug-cost related changes in medication use, including non-adherence and financial stress, were more common among beneficiaries who had multiple functional limitations and who did not have help with medications. Providing medication management support for beneficiaries with limited functional status could improve adherence and outcomes.
